# A novel role of CKIP-1 in promoting megakaryocytic differentiation

**DOI:** 10.18632/oncotarget.15619

**Published:** 2017-02-22

**Authors:** Jiao Fan, Yiwu Wang, Yuan Shen, Qingyan Liu, Rong Gao, Ya Qiu, Chanjuan Wang, Lingqiang Zhang

**Affiliations:** ^1^ State Key Laboratory of Proteomics, Beijing Proteome Research Center, Beijing Institute of Radiation Medicine, Collaborative Innovation Center for Cancer Medicine, Beijing 100850, China; ^2^ Institute of Geriatrics, Chinese PLA General Hospital, Beijing 100853, China; ^3^ Department of Infectious Diseases, Chinese PLA 532 Hospital, Anhui 242700, China; ^4^ Department of Advanced Interdisciplinary Studies, Institute of Basic Medical Sciences and Tissue Engineering Research Center, Academy of Military Medical Sciences, Beijing 100850, China; ^5^ Department of Cardiology, Chinese PLA General Hospital, Beijing 100853, China; ^6^ Department of Pharmacy, General Hospital of Air Force of Chinese PLA, Beijing 100142, China

**Keywords:** CKIP-1, megakaryocytic differentiation, PMA, transcriptional regulation, GATA-1

## Abstract

Casein kinase 2-interacting protein-1 (CKIP-1) is a known regulator of cardiomyocytes and macrophage proliferation. In this study, we showed that CKIP-1 was involved in the process of megakaryocytic differentiation. During megakaryocytic differentiation of K562 cells, CKIP-1 was dramatically upregulated and this upregulation induced by PMA was mediated through downregulation of transcription factor GATA-1. By transient transfection, oligonucleotide-directed mutagenesis and chromatin immunoprecipitation assays, we identified the transcriptional regulation of CKIP-1 by GATA-1. Overexpression of CKIP-1 initiated events of spontaneous megakaryocytic differentiation in K562 cells. Conversely, knockdown of CKIP-1 in cell lines suppressed megakaryocytic differentiation. Mechanistically, overexpression of CKIP-1 changed the expression levels of transcription factors that have been shown to be critical in erythro-megakaryocytic differentiation such as Fli-1, c-Myb and c-Myc. *In vivo* analysis confirmed that *CKIP-1^−/−^* mice had decreased number of CD41^+^ cells harvested from bone marrow, and lower platelet levels when compared to wild-type littermates. This is the first direct evidence suggesting that CKIP-1 is a novel regulator of megakaryocytic differentiation.

## INTRODUCTION

Hematopoietic stem cells (HSCs) can proliferate, self renew, and differentiate into cells of all the blood lineages to maintain hematopoiesis, which is a multistage developmental process [1]. The differentiation of hematopoietic cells is governed by a complex network of transcription factors, which ultimately determine hematopoietic cell fate leading to terminally differentiated mature blood cells [2, 3]. Megakaryocytes derive from HSCs like all blood cells. HSC gives rise to progressively committed progenitors during hematopoietic differentiation, including the common myeloid progenitor (CMP) and the megakaryocyte-erythroid progenitor (MEP), which gives rise to cells of both megakaryocytic and erythroid lineages [4]. Megakaryocyte differentiation is regulated both positively and negatively by transcription factors including GATA-binding factor 1 (GATA-1), friend leukemia virus-induced erythroleukemia-1 (Fli-1, also known as transcription factor ERGB) and c-Myb [5].

Human hematopoietic cell line K562 has severed as a model to research hematological cell differentiation, which expresses specific markers of granulocytic, monocytic, erythroid, and megakaryocytic lineages. K562 cells are regarded as pluripotent hematopoietic progenitors and can be induced to differentiate following stimulation by a variety of specific agents [6, 7]. Treatment of K562 cells with phorbol 12-myristate 13-acetate (PMA) induces typical features of megakaryocytic differentiation, including progressive polyploidization, and acquisition of lineage-specific markers [8]. The ploidy of megakaryocytes increases progressively from 8N up to 128N and directly correlates with the ability to produce platelets [9].

Previous studies reported that the PH domain-containing protein CKIP-1 (casein kinase 2-interacting protein-1, also known as PLEKHO1) played a role in the regulation of macrophage homeostasis, tumorigenesis, cardiac hypertrophy as well as cell apoptosis, cell morphology, and the actin cytoskeleton [10–18]. Our previous *in vivo* studies demonstrated that CKIP-1 depletion in mice manifested an age-dependent accumulation in bone mass due to increased osteoblast differentiation [19] and those mice were also susceptible to pressure overload-induced pathological cardiac hypertrophy involved in the HDAC4-dependent pathway [20]. We recently found that CKIP-1 was a novel regulator of macrophage homeostasis via M-CSF signaling by interacting with TRAF6 and inhibiting Akt activation [21].

This study is designed to determine whether CKIP-1 regulates hematopoietic cell differentiation. We found that CKIP-1 was upregulated during megakaryocytic differentiation of K562 cells, and the upregulation of CKIP-1 induced by PMA was mediated through downregulation of transcription factor GATA-1, which has been shown to be critical in erythro-megakaryocytic differentiation. Overexpression of CKIP-1 accelerated the megakaryocytic differentiation and knockdown of CKIP-1 attenuated the megakaryocytic differentiation in K562 cells. CKIP-1 might regulate the expression levels of certain key hematopoietic transcription factors. Furthermore, CKIP-1 was also upregulated during megakaryocytic differentiation of human CD34^+^ hematopoietic progenitor cells induced by thrombopoietin (TPO). *In vivo* analysis showed defective megakaryopoiesis and platelet production of *CKIP-1^−/−^* mice. Taken together, these data indicated a key role of CKIP-1 in megakaryocytic differentiation.

## RESULTS

### Upregulation of CKIP-1 during megakaryocytic differentiation induced by PMA

**Figure 1:**
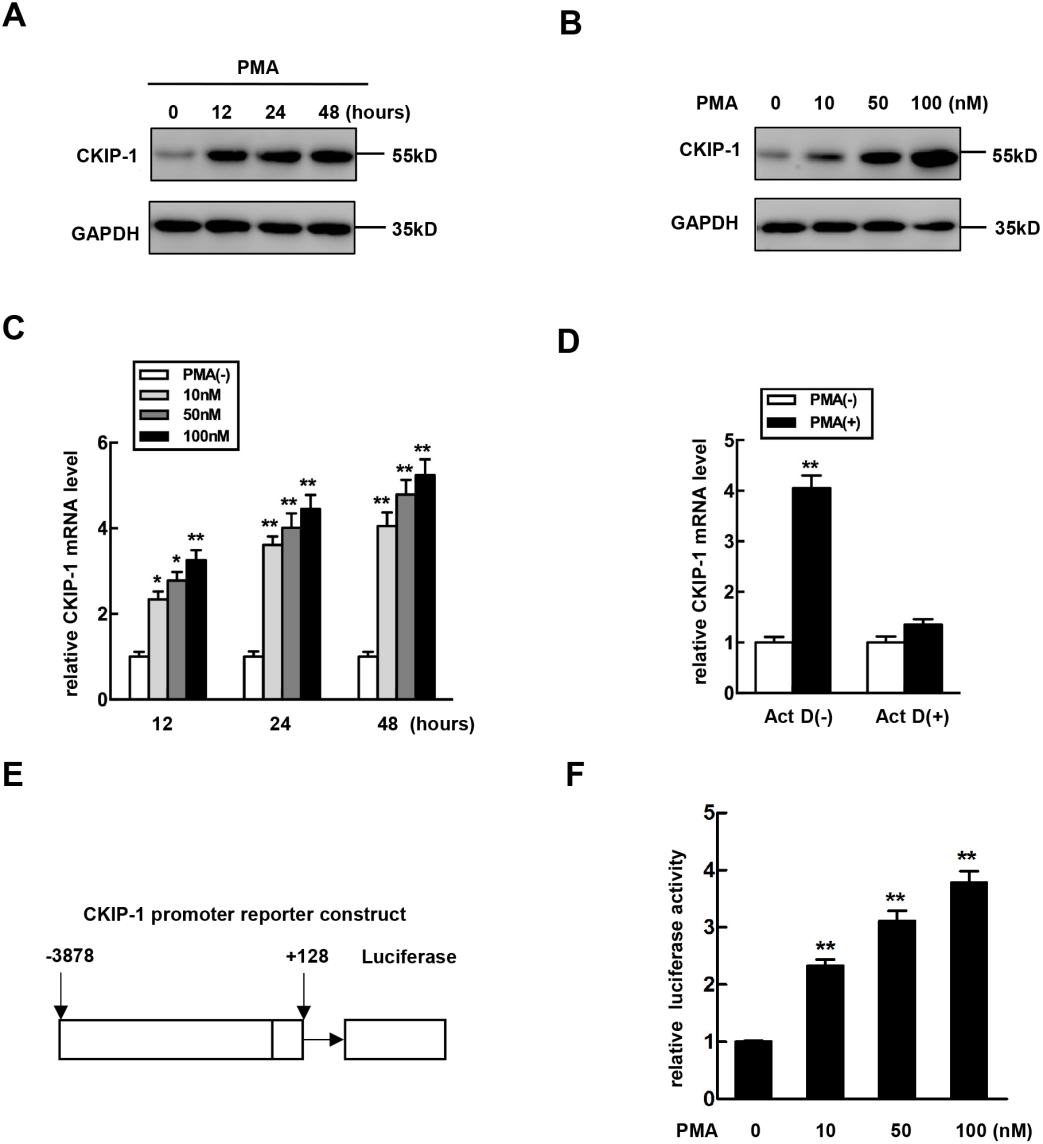
The expression of CKIP-1 is increased during megakaryocytic differentiation **A.** K562 cells were treated with 10 nM PMA for the indicated time and CKIP-1 proteins were detected by western blot. GAPDH was used as loading control. **B.** K562 cells were treated with PMA at the concentration for the indicated time and CKIP-1 proteins were detected by western blot. GAPDH was used as loading control. **C.** K562 cells were treated with PMA at the concentration indicated for the indicated time, and levels of CKIP-1 mRNA were assessed by real-time PCR. Results were expressed as fold induction compared with cells untreated with PMA and normalized to GAPDH mRNA. Data represent means ±SEM. **D.** The K562 cells were pretreated with Act D (10 μM) for 30 min prior to PMA induction. The levels of CKIP-1 mRNA were assessed by real-time PCR. The total amounts of mRNA from cells untreated with PMA were set to 1. Results were expressed as fold induction compared with cells untreated with PMA and normalized to GAPDH mRNA. Data represent means ±SEM. **E.** The -3878 to +128 region relative to transcription start site of CKIP-1 promoter was inserted in a promoter-less luciferase reporter vector pGL3-basic, resulting in CKIP-1 promoter plasmid pGL3-(−3878/+128). **F.** K562 cells were transiently transfected with pGL3-(−3878/+128) construct and then treated with PMA at the concentrations indicated for 24 hours. Then the relative luciferase activity was measured. Data represent means ±SEM.

To determine whether CKIP-1 is involved in hematopoietic differentiation, K562 cells were stimulated with PMA to promote megakaryocytic differentiation and expression levels of CKIP-1 were detected. Treatment with PMA led to a dramatic increase of CKIP-1 protein levels in K562 cells and this increase was time- and dose-dependent (Figure 1A and 1B). Then we performed real-time PCR analysis on mRNA levels of CKIP-1 and found that the upregulation of CKIP-1 induced by PMA was at least partially due to the increased accumulation of its mRNA (Figure 1C). To explore the mechanism of PMA-induced CKIP-1 upregulation, K562 cells were pretreated with actinomycin D (Act D), which has the ability to inhibit cellular transcription, and the induction of CKIP-1 mRNA expression by PMA was blocked (Figure 1D), indicating that CKIP-1 gene expression may be upregulated by PMA via transcriptional regulation. Differences in CKIP-1 expression in hematopoietic cells during differentiation suggested a potential role of CKIP-1 in megakaryocytic differentiation.

To further investigate whether the induction of CKIP-1 mRNA expression by PMA occurred through the regulation of CKIP-1 promoter, we constructed a reporter plasmid which consisted of CKIP-1 promoter region (−3878 to +128) linked to a promoter-less luciferase vector pGL3-basic (pGL3-(3878/+128)) (Figure 1E) and this reporter plasmid was transiently transfected in K562 cells to examine the effect of PMA on promoter activity. Luciferase reporter assays showed a significant increase in CKIP-1 promoter activity in a dose-dependent manner in PMA-treated cells (Figure 1F).

### Overexpression of GATA-1 reverses PMA-mediated CKIP-1 expression induction

**Figure 2:**
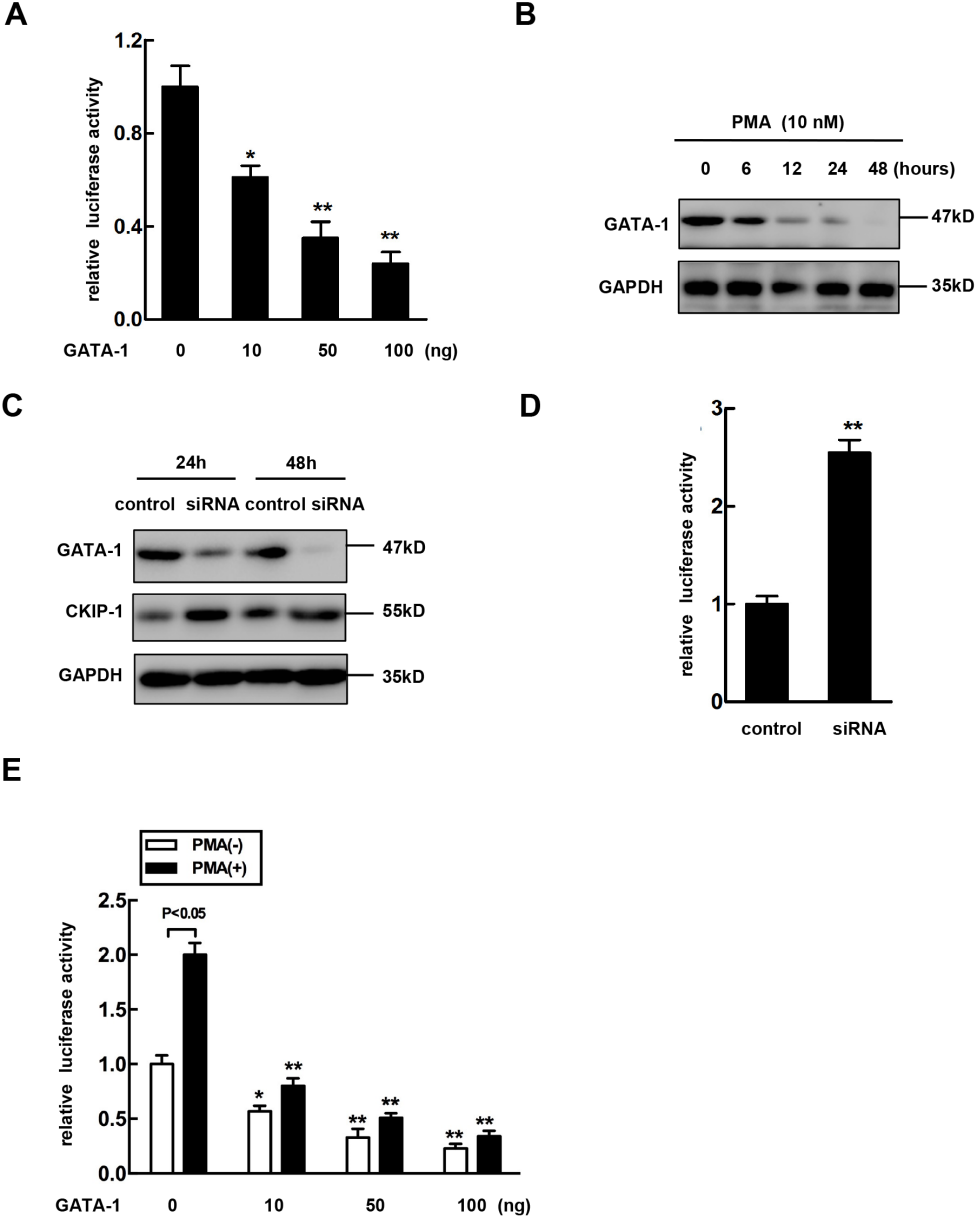
Overexpression of GATA-1 reverses PMA-mediated CKIP-1 expression induction **A.** pGL3-(−3878/+128) plasmids were cotransfected with different dose of pcDNA3.1-GATA-1 (Myc) as indicated. The assays for relative luciferase activity were performed 24 h post-transfection. Data represent means ±SEM. **B.** K562 cells were treated with 10 nM PMA for the indicated time. Then total lysates were prepared for the analysis of GATA-1 expression by western blot. GAPDH was used as loading control. **C.** A negative control siRNA or GATA-1-specific siRNA was transfected into K562 cells. The GATA-1 and CKIP-1 expression levels were detected by western blot. GAPDH was used as loading control. **D.** pGL3-(−3878/+128) plasmids were cotransfected with a negative control siRNA or GATA-1-specific siRNA into K562 cells and luciferase activity assays were performed. Data represent means ±SEM. **E.** pGL3-(−3878/+128) plasmids were cotransfected into K562 cells with different dose of GATA-1 expression vector in the absence or presence of PMA as indicated and relative luciferase activities were performed. All transfections were normalized according to Renilla-luciferase activity. Data represent means ±SEM.

Based on the previous reports suggesting that transcription factor GATA-1 is involved in erythro-megakaryocytic differentiation [22–25], we investigated the potential role of GATA-1 in the PMA-mediated regulation of CKIP-1. The pGL3-(−3878/+128) construct CKIP-1 promoter was cotransfected with GATA-1 (pcDNA3.1-GATA-1). As shown in Figure 2A, GATA-1 negatively regulated CKIP-1 promoter activity in a dose-dependent manner. Stimulation of K562 cells with PMA caused a significant reduction of GATA-1 (Figure 2B), while PMA treatment led to an increase of CKIP-1. To further investigate the effect of downregulation of GATA-1 on CKIP-1 expression, RNA interference assay was performed in K562 cells to explore whether decreased GATA-1 expression altered expression of CKIP-1. After transfection with siRNA targeted to GATA-1 into K562 cells, the levels of GATA-1 protein were reduced in a time-dependent manner and the protein levels of CKIP-1 were upregulated concomitantly with GATA-1 down-expression (Figure 2C). In K562 cells transfected with siRNA targeted to GATA-1, luciferase activity assay also confirmed the increased promoter activity of CKIP-1 (Figure 2D), suggesting that GATA-1 modulated the expression of CKIP-1.

Next we further examined the role of GATA-1 in PMA-mediated induction of CKIP-1 expression. The pcDNA3.1-GATA-1 plasmids or the control and the pGL3-(−3878/+128) CKIP-1 promoter construct were cotransfected into K562 cells. Then the luciferase activities were measured after the cells treated or untreated with PMA for 24 hours. As shown in Figure 2E, overexpression of GATA-1 provided partial but significant inhibition of PMA-mediated induction of CKIP-1 promoter activity.

### Identification of the functional GATA-1 element in the CKIP-1 promoter

**Figure 3:**
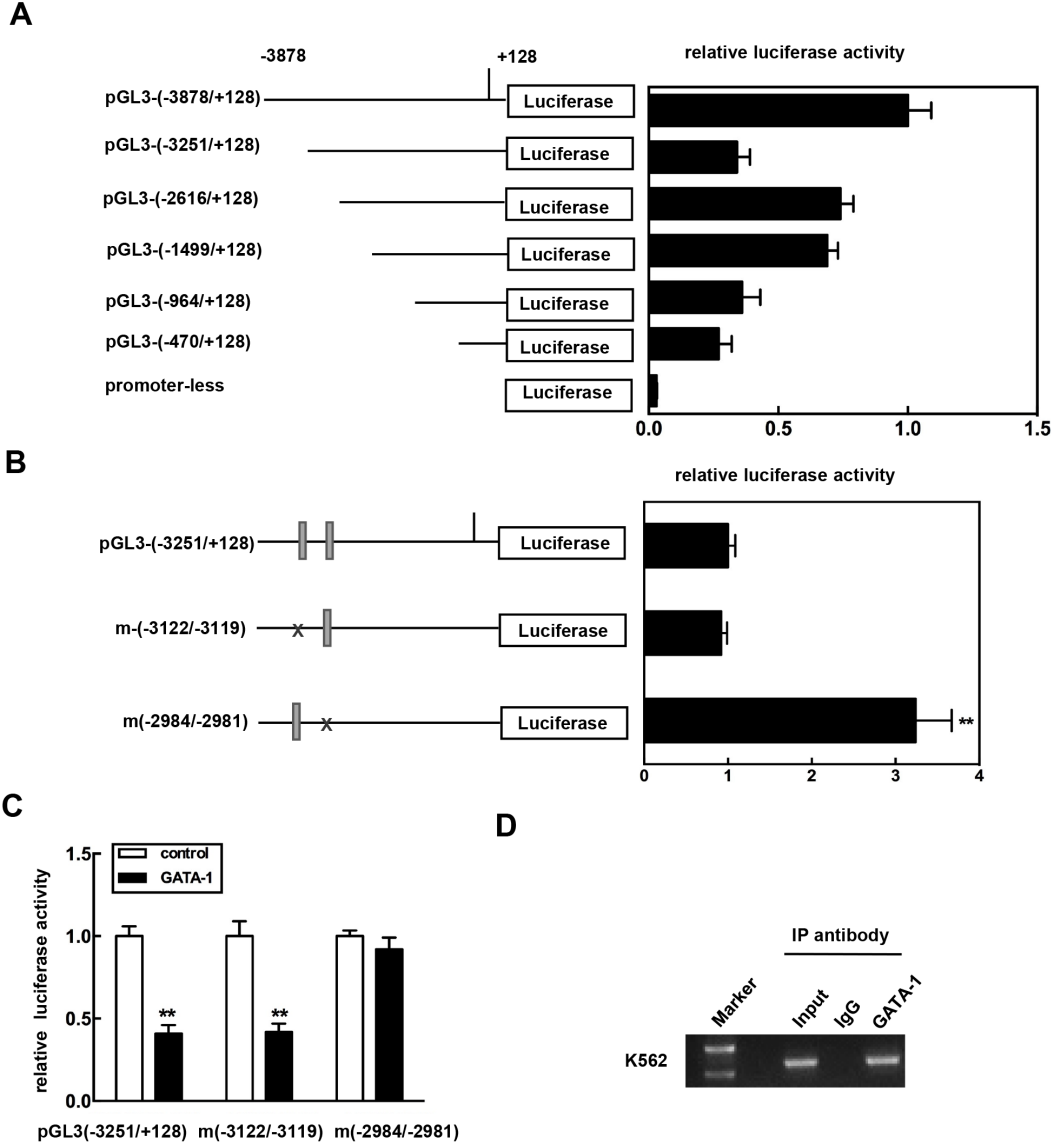
Identification of the functional GATA-1 element in the CKIP-1 promoter **A.** Schematic diagram of reporter constructs consisting of truncated CKIP-1 promoter fragments. Histogram shows the relative luciferase activity for each construct in K562 cells. Data represent means ±SEM. **B.** Reporter constructs showing the presence of wild-type or mutant GATA-1 cis element at -3122/−3119 and -2984/−2981 in the CKIP-1 promoter. Histogram shows the relative luciferase activity for each construct in K562 cells. Data represent means ±SEM. **C.** pGL3-(−3251/+128) or the mutants m(−3122/−3119) and m(−2984/−2981) was transiently cotransfected with GATA-1 expression vector pcDNA3.1-GATA-1 (Myc) into HEK 293T cells. The assays for relative luciferase activity were performed 24 h post-transfection. All transfections were normalized according to Renilla-luciferase activity. Data represent means ±SEM. **D.** Chromatin immunoprecipitation assay was carried out as described in Materials and methods. After immunoprecipitation with anti-GATA-1 antibody, DNA was purified. PCR was performed using the primers located in the region from -3010 to -2789 (P-3010/−2789). For specificity control, samples treated with unrelated IgG are also shown.

We had identified an expression pattern for CKIP-1 regulated by transcription factor GATA-1. To investigate this idea, reporter plasmids containing progressively smaller portions of the -3.8 kb fragment of CKIP-1 promoter were cloned, sequenced and the activities were analyzed in K562 cells by the luciferase reporter assays. As Figure 3A shows, activity of pGL3-(−3251/+128) was significantly lower compared to pGL3-(−2616/+128), which suggested the presence of negative regulatory cis element between −3251 bp and −2616 bp of the CKIP-1 promoter. Sequence analysis revealed that in the region of −3251 kb to −2616 kb of CKIP-1 promoter there are two consensus GATA-1 binding sites (WGATAR) [26, 27] at −3122/−3119 and -2984/−2981, respectively (Supplementary Figure 1).

To test whether one or more of these GATA-1 sites were involved in the regulation of CKIP-1 promoter, mutagenesis on the sites was performed. And then the construct mutants were transfected into K562 cells. As shown in Figure 3B, mutation of the GATA-1 site at -2984/−2981 significantly increased the promoter activity, whereas the mutation of the GATA site at -3122/−3119 had no effect on the promoter activity. Furthermore, the inhibitory effect of GATA-1 on the luciferase activity was apparently decreased with the mutation of the GATA-1 site at -2984/−2981 (Figure 3C). These results suggested that this GATA site at -2984/−2981 in the promoter was sufficient for mediating the inhibition of the CKIP-1 promoter by GATA-1.

To confirm the role for GATA-1 in regulating CKIP-1, we prepared chromatin immunoprecipitation (ChIP) assays in K562 cells to determine whether GATA-1 occupied the CKIP-1 promoter *in vivo* using the primers located in the region from -3010 to -2789 (P-3010/−2789). As shown in Figure 3D, GATA-1 was found to occupy the region of CKIP-1 promoter and the binding of GATA-1 was only detected in anti-GATA-1 precipitated chromatin fragments and not in normal IgG precipitated chromatin fragments. Collectively, these results indicated that GATA-1 bound the CKIP-1 promoter *in vivo* and GATA-1 might regulate CKIP-1 expression through a direct way.

GATA-1 has two isoforms, full length of GATA-1 and a short form of GATA-1 (GATA1s). We also detected the expression of GATA1s in K562 cells treated with or without PMA. The expression of GATA1s was much lower than that of full-length GATA-1 and was hardly detected at the protein level in K562 cells (Supplementary Figure 2).

### Overexpressing CKIP-1 in K562 cells promotes megakaryocytic differentiation

**Figure 4:**
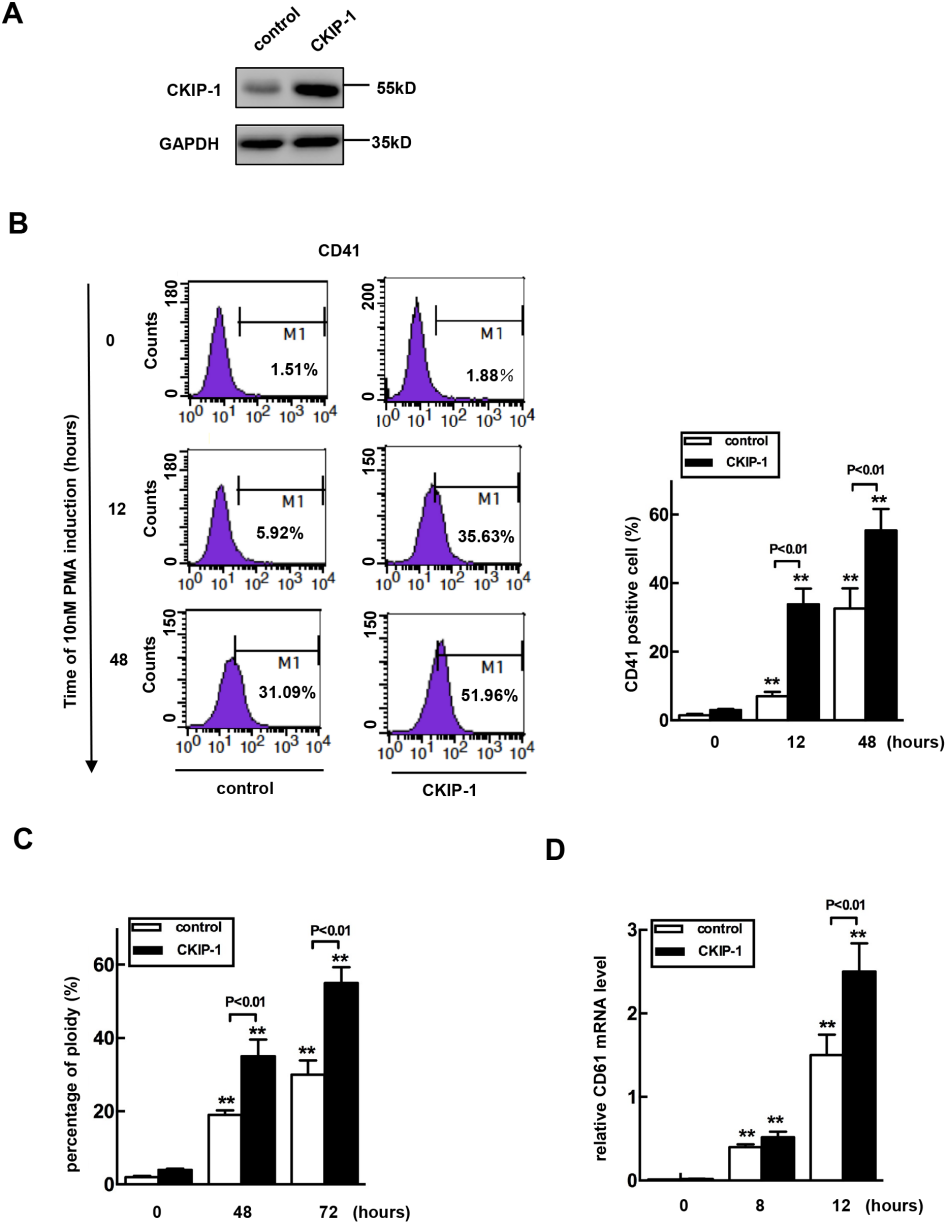
Overexpression of CKIP-1 in K562 cells induces events of early megakaryocytic differentiation **A.** K562 cells transfected with CKIP-1 and control vectors by a lentiviral system and CKIP-1 expression levels were detected by western blot. GAPDH was used as loading control. CD41^+^ cells **B.** and percentage of 4N cells **C.** were analyzed using FACS. The CD61 mRNA level was measured by real-time PCR analysis **D.** Data represent means ±SEM.

To establish the cause and effect relationship between upregulation of CKIP-1 and megakaryocytic differentiation, we constructed a CKIP-1 stably expressed K562 cell line (K562-CKIP-1). CKIP-1 expression levels were confirmed by western blot (Figure 4A). K562-CKIP-1 cells and control cells were treated with PMA for indicated lengths of time to undergo megakaryocytic differentiation and CD41 was used as a marker of mature megakaryocytes. As shown in Figure 4B, the relative numbers of CD41^+^ cells increased to 7% at 12h and to about 33% at 48h after PMA treatment in control cells. In K562-CKIP-1 cells, the relative numbers of CD41^+^ cells increased to 34% at 12h and 55% at 48h after PMA treatment. We performed ploidy analysis by flow cytometry after PMA induction and found that the percentage of 4N cells was increased in both control cells and K562-CKIP-1 cells, and the relative number of 4N cells after PMA treatment in K562-CKIP-1 cells was higher (Figure 4C). Similar results were observed that CD61 (a specific megakaryocyte marker) mRNA levels were also evaluated in K562-CKIP-1 cells than in control cells (Figure 4D). These results strongly indicated that overexpression of CKIP-1 was sufficient to induce the early events of megakaryocytic differentiation in K562 cells.

### Knockdown of CKIP-1 inhibits PMA-induced megakaryocytic differentiation in K562 cells

**Figure 5:**
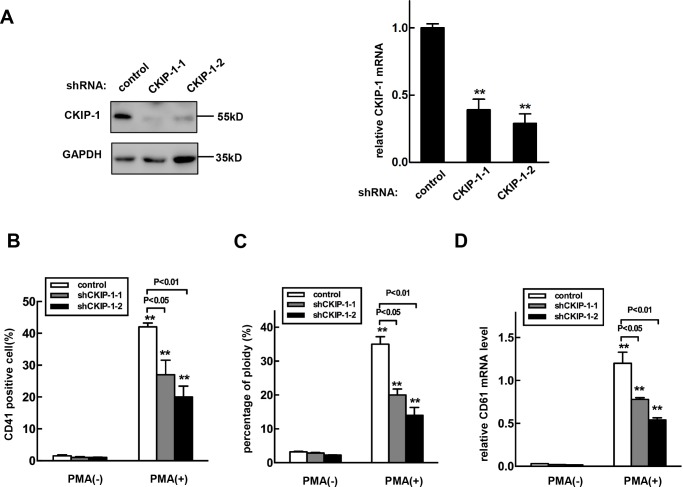
Knockdown of CKIP-1 in K562 cells inhibits PMA-induced megakaryocytic differentiation **A.** K562 cells transfected with shRNA-CKIP-1-1, -2 or shRNA- control by a lentiviral system and CKIP-1 expression levels were detected by western blot (left panel) and real-time PCR (right panel). GAPDH was used as loading control. CKIP-1 shRNA lentivirus infected-K562 cells or control K562 cells were treated with 10 nM PMA for 3 days. Then CD41^+^ cells **B.** and the percentage of 4N cells **C.** were analyzed using FACS. **D.** The CD61 mRNA levels were measured using real-time PCR analysis. Data represent means ±SEM.

We next investigated megakaryocytic differentiation in K562 cells infected with CKIP-1 shRNAs and control shRNA lentiviruses. As shown in Figure 5A, the CKIP-1 shRNA lentivirus decreased the endogenous CKIP-1 mRNA and protein levels. Knockdown of CKIP-1 led to an exactly opposite phenotype that observed with overexpression of CKIP-1. Inhibition of CKIP-1 expression in K562 cells caused reduced megakaryocytic differentiation induced by PMA with lower numbers of CD41^+^ cells (Figure 5B), 4N cells (Figure 5C) and lower expression level of CD61 mRNA (Figure 5D) as expected. Thus, knockdown of CKIP-1 prevented K562 cells from undergoing PMA-induced megakaryocytic differentiation.

### Effects of CKIP-1 overexpression on hematopoietic transcription factors

**Figure 6:**
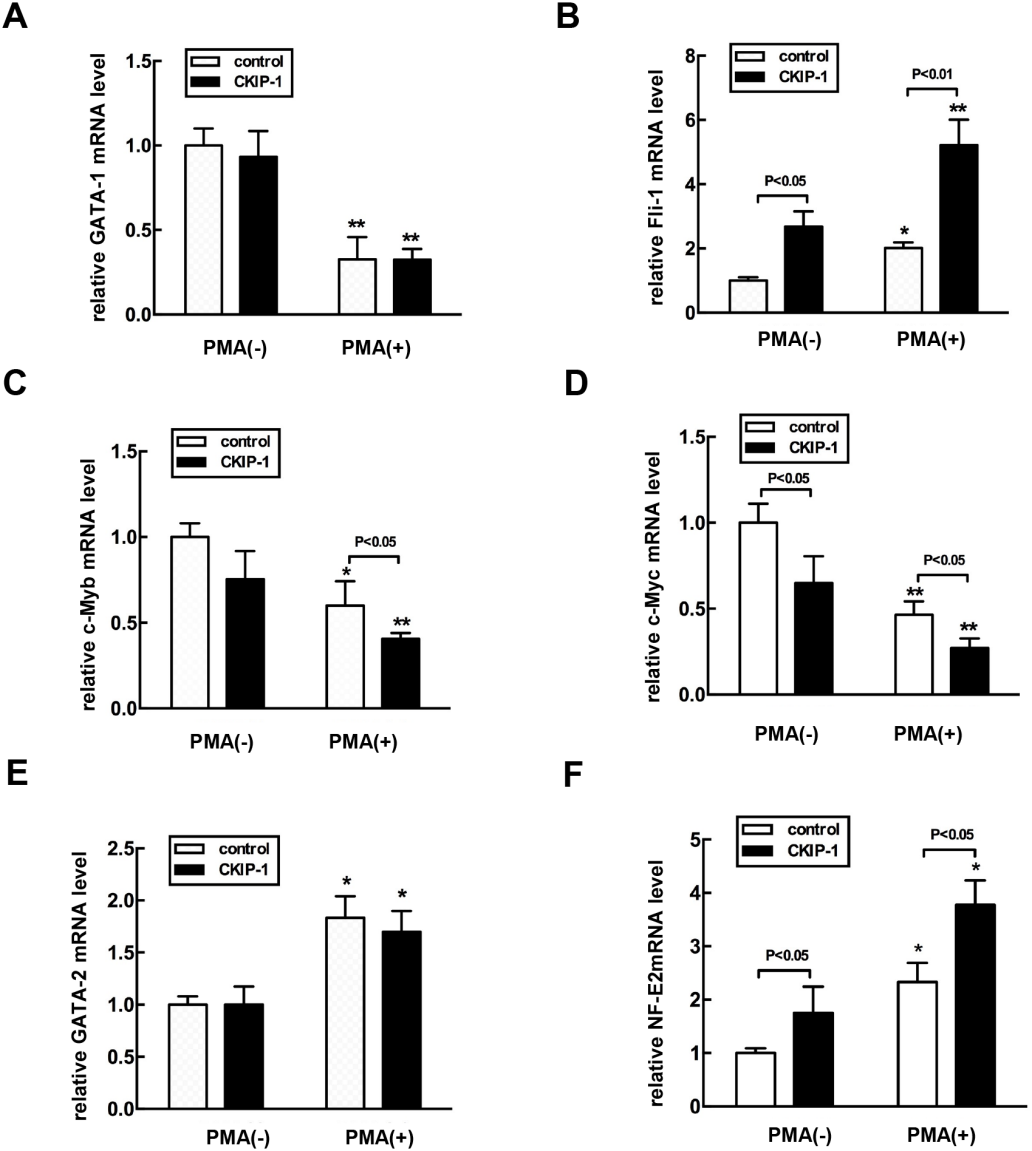
Alteration of expression levels of hematopoietic transcription factors in CKIP-1-overexpressing K562 cells **A-F.** K562-CKIP-1 cells and control cells were treated with 10 nM PMA for 72 hours. Then total RNA was extracted. Gene expression was analyzed by real-time PCR with GATA-1, Fli-1-, c-Myb-, c-Myc-, GATA-2-, and NE-F2-specific primers as described in Supplementary Table 1. Data are expressed as fold induction relative to cells at day 0 and normalized to GAPDH mRNA. Data represent means ±SEM.

Several hematopoietic transcription factors have been shown to be critical in erythro-megakaryocytic differentiation, including GATA-1, Fli-1, c-Myb, c-Myc and GATA-2 [5]. We examined whether the expression levels of these transcription factors could be modified by overexpression of CKIP-1. PMA treatment induced significant downregulation mRNA levels of GATA-1, c-Myc and c-Myb, but it upregulated GATA-2 and Fli-1 (Figures 6A–6F), which promotes megakaryocyte-lineage commitment and subsequent polyploidization [28]. In K562-CKIP-1 cells during megakaryocytic differentiation induced by PMA, the expression levels of c-Myc and c-Myb were much lower than in control cells, but the levels of Fli-1 were much higher. The mRNA levels of GATA-1 and GATA-2 mRNA in K562-CKIP-1 cells were similar to those observed in control cells. Nuclear factor erythroid 2 (NF-E2), important for regulating platelet release from mature megakaryocytes [29], showed a strong activation in K562-CKIP-1 cells. We also examined protein expression levels of these transcription factors by western blot analysis and similar results were observed (Supplementary Figure 3). These results suggested that CKIP-1 might regulate the expression levels of certain key hematopoietic transcription factors.

### Regulation of CKIP-1 during megakaryocytic differentiation of cord blood CD34^+^ cells

We investigated the expression of CKIP-1 during megakaryocytic differentiation of umbilical cord blood CD34^+^ cells. CD41 was used as a marker of mature megarkaryocytes. The CKIP-1 expression level was measured using real-time PCR and western blot analysis. In the culture system containing TPO, the number of CD41^+^ cells began to increase on day 2. On day 8 after TPO induction, the relative numbers of CD41^+^ cells increased to 35%, suggesting that CD34^+^ cells underwent megakaryocytic differentiation (Supplementary Figure 4). During the megakaryocytic differentiation of CD34^+^ cells, the CKIP-1 expression level was upregulated (Supplementary Figure 5), which suggesting that CKIP-1 might be involved in megakaryocytic differentiation of human CD34^+^ hematopoietic progenitor cells.

### Decreased number of megakaryocytes and platelets in *CKIP-1^−/−^* mice

**Figure 7:**
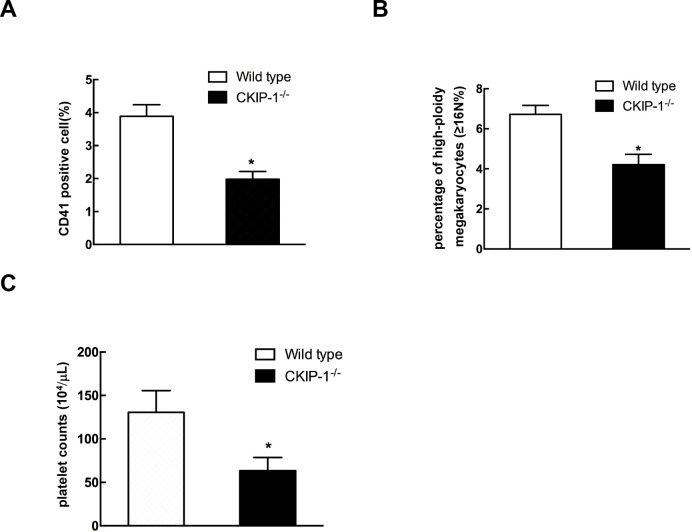
CKIP-1^−/−^ mice possess decreased numbers of CD41-positive cells and platelets CD41^+^ expression **A.** and megakaryocyte ploidy **B.** of bone marrow mononuclear cells harvested from 8–10-week-old wild-type mice and *CKIP-1^−/−^* mice from four independent experiments (n = 5). **C.** Comparison of platelets in *CKIP-1^−/−^* mice. Peripheral blood platelet counts were performed on 8-10- week-old wild-type and *CKIP-1^−/−^* mice from three independent experiments (n = 6). Data represent means ±SEM.

In order to further analyze the role of CKIP-1 during megakaryocytes development *in vivo*, we examined the expression of surface markers CD41 in CKIP-1 deficient mice. Flow cytometric analysis of bone marrow cells harvested from femurs of *CKIP-1^−/−^* mice showed a decrease in CD41-positive cells when compared to wild-type controls (Figure 7A). Furthermore, *in vivo* megakaryocyte ploidy in bone marrow from *CKIP-1^−/−^* mice was statistically significant decreased when compared to bone marrow from wild-type littermates (Figure 7B). Circulating platelet levels in *CKIP-1^−/−^* mice were decreased when compared with wild-type mice (Figure 7C), which maybe a result of reduced number of megakaryocytes.

## DISCUSSION

In this study we showed that CKIP-1 was a new regulator of megakaryocytic differentiation. CKIP-1 was originally identified as an interactive protein of casein kinase 2a13. Our previous studies demonstrate that CKIP-1 is a critical regulator of pathological cardiac hypertrophy and macrophage proliferation. Data of the present study has showed that expression of CKIP-1 was strongly increased during megakaryocytic differentiation induced by PMA in hematopoietic progenitor cell lines K562, and the PMA-induced upregulation of CKIP-1 was mediated through the downregulation of GATA-1, which is a lineage specific transcription factor. K562 cells constitutively expressed GATA-1, and GATA-1 activity was significantly downregulated during megakaryocytic differentiation induced by PMA [30]. Treatment with PMA in K562 cells showed induction of CKIP-1 transcription in a dose-dependent manner. The levels of upregulated CKIP-1 expression in K562 cells treated with PMA were concomitantly with GATA-1 down-expression and decreased GATA-1 expression by siRNA directed against GATA-1 in K562 cells increased expression of CKIP-1. The PMA-mediated expression induction of CKIP-1 was inhibited by restored expression of GATA-1 in K562 cells. Taken together, this upregulation expression of CKIP-1 mediated by PMA may be linked to transcriptional control by GATA-1. Our previous studies have identified CKIP-1 as a novel substrate of GSK3β and GSK3β inhibits CKIP-1 through phosphorylation followed by ubiquitination and proteasomal degradation [15]. To the best of knowledge, this is the first report of transcriptional regulation of CKIP-1 and GATA-1 may be the first identified transcription factor of CKIP-1. Compared with full-length GATA-1, GATA1s has a reduced transactivation potential because of the absence of the N-terminal transactivation domain [31] and this isoform was linked to a highly informative pair of disorders—transient abnormal myelopoiesis (TAM) [32] and acute megakaryoblastic leukaemia (AMKL) [31, 33]. In our present studies, we mainly focused on relationship between full length of GATA-1 and CKIP-1. However, it is of great value that to investigate the GATA1s-CKIP-1 regulation and we will continue to explore this question in the future.

Next we hypothesized that CKIP-1 played a role in megakaryocytic differentiation. K562 cell lines, which are widely used models to study hematopoietic differentiation, have an advantage over primary cells of being homogeneous and easily cultured. Overexpression of CKIP-1 in K562 cells was sufficient to induce enhanced megakaryocytic differentiation. The numbers of 4N cells, CD41^+^ cells and the level of CD61 mRNA were significantly increased. Downregulation of CKIP-1 in K562 cells produced an exactly opposite phenotype.

To gain insight into how CKIP-1 mediated megakaryopoiesis at the molecular level, we focused on the regulation of hematopoietic transcription factors. The expression levels of c-Myc, c-Myb and Fli-1 were changed by overexpression of CKIP-1 in K562 cells. c-Myc has been reported to be involved in hematopoietic differentiation. *c-Myc^−/−^* mice show increased numbers of megakaryocytic progenitors and mature megakaryocytes, which suggests an important role of c-Myc in control the cell fate of megakaryocyte-erythrocyte progenitors [34]. This phenotype is very similar to that of CKIP-1 overexpressing K562 cells. Overexpression of CKIP-1 leads to accelerated megakaryocytic differentiation, together with reduced c-Myc expression, suggesting that CKIP-1 plays a role opposite to that of c-Myc in hematopoietic differentiation. Overexpression of CKIP-1 also modified the expression of transcription factors c-Myb and Fli-1. In human CD34^+^ hematopoietic progenitor cells, c-Myb silencing led to increased commitment capacity toward megakaryocyte lineages but impaired erythroid differentiation reported by previous studies [35], suggesting that c-Myb plays a positive role in erythroid differentiation. Fli-1 induces megakaryocytic differentiation but suppresses erythroid differentiation in human hematopoietic cells [36, 37]. Our data demonstrated that overexpression of CKIP-1 inhibited the expression of c-Myb and enhanced the expression of Fli-1, which suggests that CKIP-1 may play the converse role on erythroid and megakaryocytic differentiation associated with alterations of transcription factors such as upregulation of the megakaryocytic related genes and repression of the genes related to erythroid differentiation. The role of CKIP-1 in erythroid differentiation need to be further investigated. CKIP-1 significantly enhanced the level of NF-E2, important for regulating platelet release from mature megakaryocytes [29], which is in line with the decreased production of platelet levels in *CKIP-1^−/−^* mice. The exact molecular mechanisms of how CKIP-1 regulated the expression of transcription factors remain to be elucidated.

Noted the leukemia origin of K562 cells, we also detected the expression of CKIP-1 during megakaryocytic differentiation of human CD34^+^ hematopoietic progenitor cells, and upregulation of CKIP-1 expression level was observed. CKIP-1 knockout mice showed decreased numbers of bone marrow-derived CD41^+^ cells and decreased circulating platelet levels when compared to wild-type littermates. However, due to the deficiency of CKIP-1 in all cells in knockout mice and CKIP-1 was known to regulate macrophage proliferation, osteoporosis and many other processes, it is possible that CKIP-1 depletion in other cells other than hematopoietic cells participate the differentiation of megakaryocytes, which need to be further investigated.

Taken together, we provided the first line of evidence that CKIP-1 regulated megakaryocytic differentiation and CKIP-1 knockout mice showed defective megakaryopoiesis and platelet production, suggesting a novel role for CKIP-1 in hematopoietic differentiation.

## MATERIALS AND METHODS

### Cell culture and reagents

Human hematopoietic cell line K562 was cultured in RPMI1640 medium (Gibco), supplemented with 10% fetal bovine serum (FBS) and 1% penicillin and streptomycin sulfate. Cells were incubated at 37°C in a humidified chamber with 5% CO2. When megakaryocytic differentiation was induced, K562 cells were treated with PMA (Sigma-Aldrich) at the indicated concentration for the indicated time. Megakaryocytic differentiation was assessed by analyzing CD41 positive cells and assessing the relative number of 4N cells [38] in K562 cells, and the CD61 mRNA level was detected by real-time PCR. Act D was purchased from Sigma-Aldrich.

### Western blotting analysis

Cells were collected and lysed in RIPA buffer with proteinase inhibitors. Immunoblot was performed using the standard protocol. Primary antibodies were used according to the manufacturer’s specifications: anti-CKIP-1 (D20, Santa Cruz), anti-GATA-1 (M20, Santa Cruz), anti-GATA-2 (ab109241, Abcam), anti-Fli-1(ab133485, Abcam), anti-c-Myb (ab109127, Abcam), anti-c-Myc (ab32072, Abcam), anti-NF-E2 (ab140598, Abcam) and anti-GAPDH (MBL).

### Quantitative real-time PCR

Total RNA was isolated using TRIzol reagent (Invitrogen) and reverse-transcribed using ReverTra Ace (Toyobo). Quantitative real-time PCR was performed using gene-specific primers (Supplementary Table 1) in an iQ5 real-time PCR system (Bio-rad) using Realtime PCR Master Mix (Toyobo). Relative expression level of target genes was normalized using GAPDH mRNA.

### Reporter constructs and site-directed mutagenesis

Human genomic DNA was amplified by PCR using a common reverse primer complimentary to DNA sequence 128 base pairs (bp) upstream of the translation start site in the CKIP-1 gene, and various forward primers truncated at −3878, −3251, −2616, −1499, −964 and −470 (Supplementary Table 1). These DNA fragments were then cloned into a promoter-less luciferase vector pGL3-basic (Promega) via SacI and Xhol cleavage sites. Oligonucleotide-directed mutagenesis with the overlap extension PCR amplification method was used to mutate the two GATA-1 binding sites in the context of pGL3-(−3251/+128) at −3122/−3119 (TTTTATCTCT→TTTGCCATCT) and -2984/−2981 (TCTTATCCTC→TCTGCCACTC), and verified by DNA sequencing.

### Transfection and luciferase assay

Cells (2×10^5^ cell per well in 6-well plates) were transfected with Lipofectamine™ 2000 transfection reagent (Invitrogen) following the manufacturer's instructions. Briefly, cells were transfected with 2μg of each construct reporter plasmid and pRL-TK vector was cotransfected which led to the constitutive expression of Rennila luciferase. Luciferase activity was assessed by Dual-Luciferase® Reporter Assay System (Promega) 24 hours after transfection according to the manufacturer’s instructions.

### Viral infection

CKIP-1 cDNAs were inserted into murine stem cell virus (MSCV)-IRESGFP or (MSCV)-IRES-Puro vector for overexpression assay. CKIP-1 shRNAs were inserted into U6-Puro-GFP vector for knockdown assays. CKIP-1-lentivivral shRNA-1 (5′-CCT GAG TGA CTA TGA GAA GTT-3′), CKIP-1-lentivivral shRNA-2 (5′-AGT GCG AAG AGC TCC GGA AAT-3′), control shRNA (5′-TTC TCC GAA CGT GTC ACG T-3′). Lentivivrals were transfected with packing plasmids into 293FT cells for 2 days, and virus particles were used to infect K562 cells. Selections were carried out if necessary by culturing in medium containing 2 μg/ml puromycin for 2 days.

### Small interfering RNA

Small interfering RNA (siRNA) targeted to human GATA-1 gene was synthesized by Shanghai GenePharma Co, Ltd. GATA-1 siRNA: GCG CCU GAU UGU CAG UAA ATT.

### Chromatin immunoprecipitation assay

ChIP assays were performed as described [39]. K562 cells were used for the crosslinking with formaldehyde. For the immunoprecipitation, normal IgG or anti-GATA-1-antibody (Santa Cruz) was added and incubated at 4°C overnight. Immunocomplexes were extracted and crosslinking was reversed at 65°C overnight. Protein was digested with proteinase K at 45°C for 2 hours. DNA was purified by phenol/chloroform extraction and ethanol precipitation. Primer sequences are listed in supplementary Table 1.

### Isolation and culture conditions of human cord blood CD34^+^ cells and induction of differentiation

The umbilical cord blood samples were obtained from volunteers after informed consent in accordance with the institutional guidelines of the committee on Human Investigation. Human CD34^+^ cells were isolated by positive selection from mononuclear cells with anti-CD34 microbeads according to the manufacturer′s instructions (MACS, Miltenyi Biotec GmbH). To induce megakaryocytic differentiation, the CD34^+^ cells were cultured in StemSpan serumfree medium (SFEM) (StemCell Technologies) with 50 ng/mL stem cell factor (SCF), 50 ng/mL TPO, 10 ng/mL interleukin-6 (IL-6), 10 ng/mL IL-3 (R&D Systems Inc).

### Mice

*CKIP-1^−/−^* mice generated as previously described [19] were bred and housed in specific pathogen-free facilities. All experimental procedures in mice were approved by the Laboratory Animal Center of Chinese Academy of Military Medical Sciences.

### Platelet analysis

Mice were anesthetized and peripheral blood was collected in ethylenediaminetetraacetic acid (EDTA)-covered Microvette collection tubes for complete blood cell (CBC) analysis on fully Automatic hematology analyzer.

### Flow cytometric analysis

Bone marrow cells isolated from wild-type and *CKIP-1^−/−^* mice were made into single-cell suspensions in FACS buffer (cold PBS supplemented with 1% FBS). Cells were stained on ice with CD41-PE (BD Pharmigen) or control antibody for 30 min. The stained cells were washed twice in FACS buffer and FACS analyses were done on FACSCalibur (BD Biosciences). Data were analyzed with the FlowJo software (Treestar). For ploidy analysis, murine megakaryocyte cells labeled with anti-CD41-PE were fixed for 15 min at room temperature in 0.5% paraformaldehyde in PBS, permeabilized in 70% ethanol, and stained with propidium iodide (PI).

### Statistical analysis

Data are presented as mean ±SEM. The statistical significance of differences was evaluated with the Student's t test or a one-way analysis of variance (ANOVA). Significance was accepted at the level of p < 0.05. In all experiments, the number of asterisks represent the following: ***p < 0.001, **p < 0.01, *p < 0.05.

## SUPPLEMENTARY FIGURES AND TABLE



## References

[1] Iwasaki H, Akashi K (2007). Hematopoietic developmental pathways: on cellular basis. Oncogene.

[2] Cantor AB, Orkin SH (2001). Hematopoietic development: a balancing act. Curr Opin Genet Dev.

[3] Shivdasani RA, Orkin SH (1996). The transcriptional control of hematopoiesis. Blood.

[4] Szalai G, LaRue AC, Watson DK (2006). Molecular mechanisms of megakaryopoiesis. Cell Mol Life Sci.

[5] Dore LC, Crispino JD (2011). Transcription factor networks in erythroid cell and megakaryocyte development. Blood.

[6] Leary JF, Ohlsson-Wilhelm BM, Giuliano R, LaBella S, Farley B, Rowley PT (1987). Multipotent human hematopoietic cell line K562: lineage-specific constitutive and inducible antigens. Leuk Res.

[7] Sutherland JA, Turner AR, Mannoni P, McGann LE, Turc JM (1986). Differentiation of K562 leukemia cells along erythroid, macrophage, and megakaryocyte lineages. J Biol Response Mod.

[8] Ravid K, Lu J, Zimmet JM, Jones MR (2002). Roads to polyploidy: the megakaryocyte example. J Cell Physiol.

[9] Mattia G, Vulcano F, Milazzo L, Barca A, Macioce G, Giampaolo A, Hassan HJ (2002). Different ploidy levels of megakaryocytes generated from peripheral or cord blood CD34+ cells are correlated with different levels of platelet release. Blood.

[10] Bosc DG, Graham KC, Saulnier RB, Zhang C, Prober D, Gietz RD, Litchfield DW (2000). Identification and characterization of CKIP-1, a novel pleckstrin homology domain-containing protein that interacts with protein kinase CK2. J Biol Chem.

[11] Safi A, Vandromme M, Caussanel S, Valdacci L, Baas D, Vidal M, Brun G, Schaeffer L, Goillot E (2004). Role for the pleckstrin homology domain-containing protein CKIP-1 in phosphatidylinositol 3-kinase-regulated muscle differentiation. Mol Cell Biol.

[12] Canton DA, Olsten ME, Kim K, Doherty-Kirby A, Lajoie G, Cooper JA, Litchfield DW (2005). The pleckstrin homology domain-containing protein CKIP-1 is involved in regulation of cell morphology and the actin cytoskeleton and interaction with actin capping protein. Mol Cell Biol.

[13] Zhang L, Xing G, Tie Y, Tang Y, Tian C, Li L, Sun L, Wei H, Zhu Y, He F (2005). Role for the pleckstrin homology domain-containing protein CKIP-1 in AP-1 regulation and apoptosis. EMBO J.

[14] Canton DA, Olsten ME, Niederstrasser H, Cooper JA, Litchfield DW (2006). The role of CKIP-1 in cell morphology depends on its interaction with actin-capping protein. J Biol Chem.

[15] Zhang L, Tie Y, Tian C, Xing G, Song Y, Zhu Y, Sun Z, He F (2006). CKIP-1 recruits nuclear ATM partially to the plasma membrane through interaction with ATM. Cell Signal.

[16] Zhang L, Tang Y, Tie Y, Tian C, Wang J, Dong Y, Sun Z, He F (2007). The PH domain containing protein CKIP-1 binds to IFP35 and Nmi and is involved in cytokine signaling. Cell Signal.

[17] Xi S, Tie Y, Lu K, Zhang M, Yin X, Chen J, Xing G, Tian C, Zheng X, He F, Zhang L (2010). N-terminal PH domain and C-terminal auto-inhibitory region of CKIP-1 coordinate to determine its nucleus-plasma membrane shuttling. FEBS Lett.

[18] Tokuda E, Fujita N, Oh-hara T, Sato S, Kurata A, Katayama R, Itoh T, Takenawa T, Miyazono K, Tsuruo T (2007). Casein kinase 2-interacting protein-1, a novel Akt pleckstrin homology domain-interacting protein, down-regulates PI3K/Akt signaling and suppresses tumor growth *in vivo*. Cancer Res.

[19] Lu K, Yin X, Weng T, Xi S, Li L, Xing G, Cheng X, Yang X, Zhang L, He F (2008). Targeting WW domains linker of HECT-type ubiquitin ligase Smurf1 for activation by CKIP-1. Nat Cell Biol.

[20] Ling S, Sun Q, Li Y, Zhang L, Zhang P, Wang X, Tian C, Li Q, Song J, Liu H, Kan G, Cao H, Huang Z (2012). CKIP-1 inhibits cardiac hypertrophy by regulating class II histone deacetylase phosphorylation through recruiting PP2A. Circulation.

[21] Zhang L, Wang Y, Xiao F, Wang S, Xing G, Li Y, Yin X, Lu K, Wei R, Fan J, Chen Y, Li T, Xie P (2014). CKIP-1 regulates macrophage proliferation by inhibiting TRAF6-mediated Akt activation. Cell Res.

[22] Migliaccio AR, Rana RA, Vannucchi AM, Manzoli FA (2005). Role of GATA-1 in normal and neoplastic hemopoiesis. Ann N Y Acad Sci.

[23] Orkin SH, Shivdasani RA, Fujiwara Y, McDevitt MA (1998). Transcription factor GATA-1 in megakaryocyte development. Stem Cells.

[24] Dai W, Murphy MJ (1993). Downregulation of GATA-1 expression during phorbol myristate acetate-induced megakaryocytic differentiation of human erythroleukemia cells. Blood.

[25] Shimamoto T, Ohyashiki JH, Ohyashiki K, Toyama K (1995). [The expression pattern of transcription factors (GATA, SCL) and biological characteristics in various leukemia cells]. Rinsho Ketsueki.

[26] Cheng Y, King DC, Dore LC, Zhang X, Zhou Y, Zhang Y, Dorman C, Abebe D, Kumar SA, Chiaromonte F, Miller W, Green RD, Weiss MJ (2008). Transcriptional enhancement by GATA1-occupied DNA segments is strongly associated with evolutionary constraint on the binding site motif. Genome Res.

[27] Evans T, Reitman M, Felsenfeld G (1988). An erythrocyte-specific DNA-binding factor recognizes a regulatory sequence common to all chicken globin genes. Proc Natl Acad Sci U S A.

[28] Raslova H, Roy L, Vourc’h C, Le Couedic JP, Brison O, Metivier D, Feunteun J, Kroemer G, Debili N, Vainchenker W (2003). Megakaryocyte polyploidization is associated with a functional gene amplification. Blood.

[29] Shivdasani RA, Orkin SH (1995). Erythropoiesis and globin gene expression in mice lacking the transcription factor NF-E2. Proc Natl Acad Sci U S A.

[30] Li CY, Fang F, Xu WX, Xu CW, Zhan YQ, Wang ZD, Ding YL, Li YH, Sun HB, Yang XM (2008). Suppression of EDAG gene expression by phorbol 12-myristate 13-acetate is mediated through down-regulation of GATA-1. Biochim Biophys Acta.

[31] Wechsler J, Greene M, McDevitt MA, Anastasi J, Karp JE, Le Beau MM, Crispino JD (2002). Acquired mutations in GATA1 in the megakaryoblastic leukemia of Down syndrome. Nature genetics.

[32] Greene ME, Mundschau G, Wechsler J, McDevitt M, Gamis A, Karp J, Gurbuxani S, Arceci R, Crispino JD (2003). Mutations in GATA1 in both transient myeloproliferative disorder and acute megakaryoblastic leukemia of Down syndrome. Blood Cells Mol Dis.

[33] Zipursky A (2003). Transient leukaemia--a benign form of leukaemia in newborn infants with trisomy 21. Br J Haematol.

[34] Guo Y, Niu C, Breslin P, Tang M, Zhang S, Wei W, Kini AR, Paner GP, Alkan S, Morris SW, Diaz M, Stiff PJ, Zhang J (2009). c-Myc-mediated control of cell fate in megakaryocyte-erythrocyte progenitors. Blood.

[35] Bianchi E, Zini R, Salati S, Tenedini E, Norfo R, Tagliafico E, Manfredini R, Ferrari S (2010). c-myb supports erythropoiesis through the transactivation of KLF1 and LMO2 expression. Blood.

[36] Athanasiou M, Mavrothalassitis G, Sun-Hoffman L, Blair DG (2000). FLI-1 is a suppressor of erythroid differentiation in human hematopoietic cells. Leukemia.

[37] Athanasiou M, Clausen PA, Mavrothalassitis GJ, Zhang XK, Watson DK, Blair DG (1996). Increased expression of the ETS-related transcription factor FLI-1/ERGB correlates with and can induce the megakaryocytic phenotype. Cell Growth Differ.

[38] Rubin CI, French DL, Atweh GF (2003). Stathmin expression and megakaryocyte differentiation: a potential role in polyploidy. Exp Hematol.

[39] Letting DL, Rakowski C, Weiss MJ, Blobel GA (2003). Formation of a tissue-specific histone acetylation pattern by the hematopoietic transcription factor GATA-1. Mol Cell Biol.

