# Supplementary material for: A novel role of CKIP-1 in promoting megakaryocytic differentiation
Source: Oncotarget. 2017 Feb 22;8(18):30138–50. doi: 10.18632/oncotarget.15619 (PMC5444732; doi:10.18632/oncotarget.15619)
Supplement: Supplementary file 1 [file oncotarget-08-30138-s001.pdf]

A novel role of CKIP-1 in promoting megakaryocytic differentiation

SUPPLEMENTARY FIGURES AND TABLE

-3300	ACTTTAATTA	AGGTCTTGTA	TGCTTTTAAT	AATCCTCTCC	CCCATAGGTG
-3250	GAAATGCCCT	GGTCAAAACC	TCTGTGTTCC	ATTTACTCCA	CAAAAGCCAA
-3200	GGATATTTGT	TTTTAGACAA	CTGTGTTTGA	ATAACAGTTT	CTGTCCAGTA
-3150	AACTTGCAAG	CAGCAGTTGA	CAAGTTTT<u>TA</u> (GATA-1, -3122/-3119)	<u>TC</u>TCTAATAG	TTCCCTTCTT
-3100	TCTCCCTTTA	GTGAAGTTAT	AAAATATTCC	GTTATAGAAT	ACAGCTACTA
-3050	GCAGTCGCGT	CTTATCCTCA	GATGTTGTTA	GAGAGGTACC	CCCATCATGA
-3000	GGTCCTCCCA	GTGTCT<u>TATC</u> (GATA-1, -2984/-2981)	TTCACCACAG	ACAGAAACTG	GCTAGAGTGA
-2950	TTTCTGTGGT	TTATGTGCGT	GTAGTGTCAA	GCTCACTGAG	GAGCGCTGAG
-2900	GAGACAGCGC	TATAAAAATG	TGTATAGGAA	CACATACGTT	CTCAGATTTC
-2850	TCACTTTAGA	CATTGGTCTTT	GGAAAACAGT	CCCACCTACA	TCCCAATTCA

Supplementary Figure 1: DNA sequence of the promoter of the human CKIP-1 gene. The putative consensus binding sites for GATA-1 are underlined.

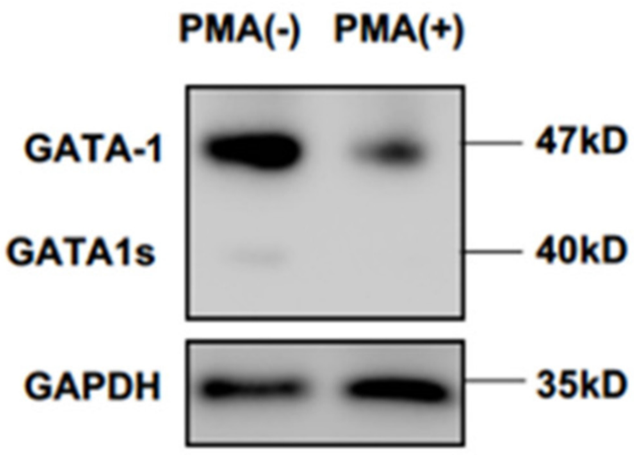

Supplementary Figure 2: Expression levels of GATA-1 and GATA1s in K562 cells treated with or without PMA for 12 hours detected by western blot using GATA-1 antibody (M20, Santa Cruz). The predicted size of GATA-1 is 47 kDa and GATA1s is 40 kDa. GAPDH was used as loading control.

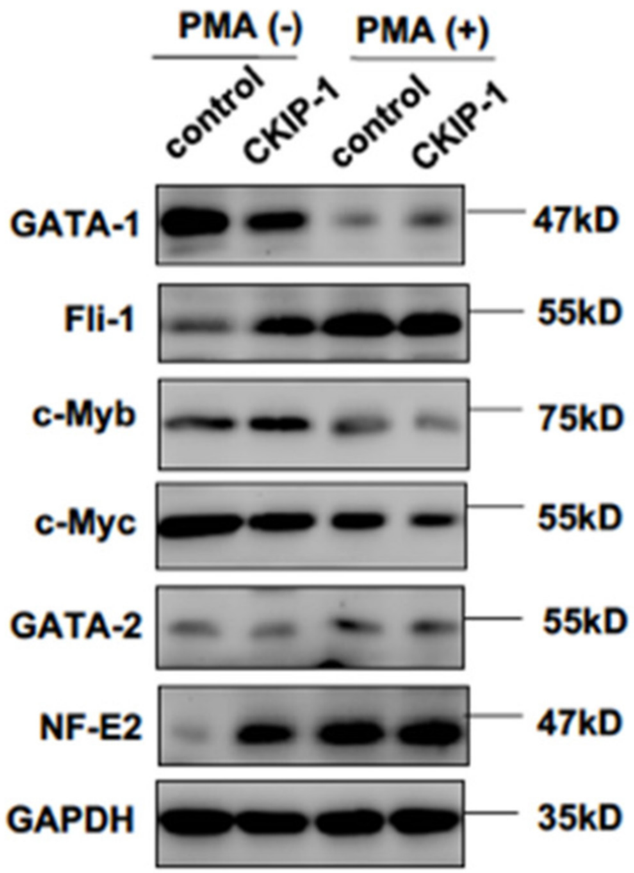

Supplementary Figure 3: K562-CKIP-1 cells and control cells were treated with or without PMA. Then protein levels of GATA-1, Fli-1, c-Myb, c-Myc, GATA-2, and NE-F2 were detected by western blot. GAPDH was used as loading control.

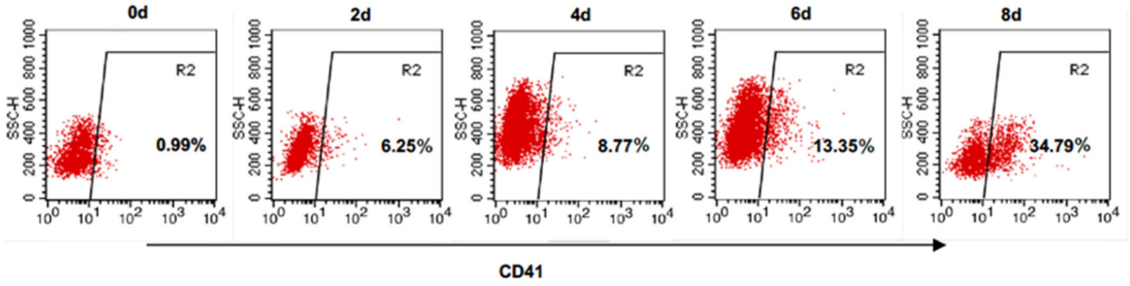

Supplementary Figure 4: Megakaryocytic differentiation of CD34⁺ cells. Human cord blood CD34⁺ cells were cultured in the presence of TPO for the indicated time and then cells were stained with PE-CD41 antibody for flow cytometry analysis.

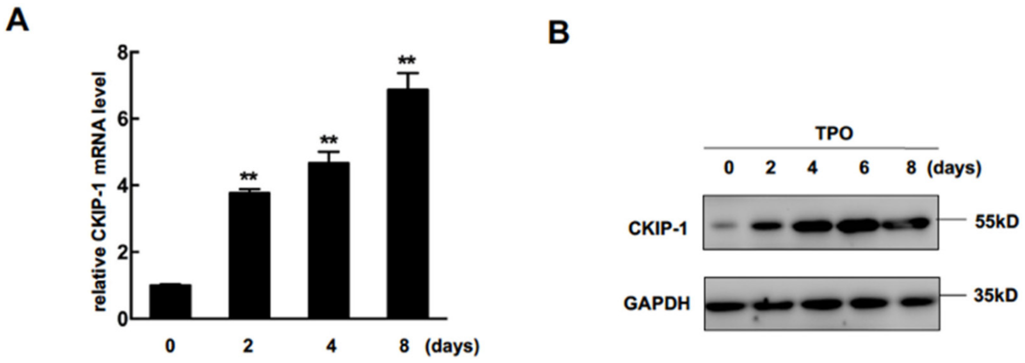

Supplementary Figure 5: Human cord blood CD34⁺ cells were cultured in the presence of TPO for the indicated time. **A.** Levels of CKIP-1 mRNA were assessed by real-time PCR. Data represent means ±SEM. **B.** Levels of CKIP-1 protein were detected by western blot. GAPDH was used as loading control.

Supplementary Table 1: Sequence of Primers used in the present study

CKIP-1 Luc reporter constructs	*Forward*	*Reverse*
−3878/+128	AACTCCTGACCTCGTGATCC	TCCCCTTCTTTGTAGCTCCG
−3251/+128	GGAAATGCCCTGGTCAAAAC	
−2616/+128	GACAGGCAGGGAAGGTTCT	
−1499/+128	ACTGGAAAAGAGAGGGAGCC	
−964/+128	GGCTTCTCTAGTCAGGCAT	
−470/+128	AATTCCTGATGCGGGTTGTG	
**ChIP assay**	***Forward***	***Reverse***
−3010/-2789	CCCATCATGAGGTCCTCCC	CTCATGGCACCTGAATTGGG
**RT-PCR**	***Forward***	***Reverse***
CKIP-1	ACCCGAGCCAAGAACCGTAT	TGGAAGCCACAGCCATTAGG
CD61	TATAGCATTGGACGGAAGGC	GACCTCATTGTTGAGGCAGG
Fil-1	CCCACCAGCAGAAGGTGAAC	ATGCGGCTCCAAAGAAGCT
c-Myb	TTCTGAAGCACAAAATGTCTC	CCCACATAATGGTAGCACCTG
c-Myc	CGTCTCCACACATCAGAGCAC	GCAGCAGGATAGTCCTT
GATA-1	GCACCAACTGCCAGACGACC	CAGATGCCTTGCGGTTTCGA
GATA-2	ATCCACCCTTCCTCCAGTCT	CGGGAGCCAAGAGTATGTTC
NE-F2	TGGGACCATCTTCCTTGTG	TTGCCATTGTCATCCTCTTCT
GAPDH	ATGGGGAAGGTGAAGGTCGG	GACGGTGCCATGGAATTTGC
